# Ethnic variations in pathways to acute care and compulsory detention for women experiencing a mental health crisis

**DOI:** 10.1177/0020764010382369

**Published:** 2012-01

**Authors:** Caroline Lawlor, Sonia Johnson, Laura Cole, Louise M. Howard

**Affiliations:** 1 University College London, London, UK; 2 Institute of Psychiatry, London, UK

**Keywords:** ethnicity, pathway to care, compulsory admission, women’s mental health

## Abstract

**Background**: Much recent debate on excess rates of compulsory detention and coercive routes to care has focused on young black men; evidence is less clear regarding ethnic variations among women and factors that may mediate these.

**Aim**: To explore ethnic variations in compulsory detentions of women, and to explore the potential role of immediate pathways to admission and clinician-rated reasons for admission as mediators of these differences.

**Method**: All women admitted to an acute psychiatric inpatient ward or a women’s crisis house in four London boroughs during a 12-week period were included. Data were collected regarding their pathways to care, clinician-rated reasons for admission, hospital stays, and social and clinical characteristics.

**Results**: Two hundred and eighty seven (287) women from white British, white other, black Caribbean, black African and black other groups were included. Adjusting for social and clinical characteristics, all groups of black patients and white other patients were significantly more likely to have been compulsorily admitted than white British patients; white British patients were more likely than other groups to be admitted to a crisis house and more likely than all the black groups to be admitted because of perceived suicide risk. Immediate pathways to care differed: white other, black African and black other groups were less likely to have referred themselves in a crisis and more likely to have been in contact with the police. When adjustment was made for differences in pathways to care, the ethnic differences in compulsory admission were considerably reduced.

**Discussion**: There are marked ethnic inequities not only between white British and black women, but also between white British and white other women in experiences of acute admission. Differences between groups in help-seeking behaviours in a crisis may contribute to explaining differences in rates of compulsory admission.

## Introduction

Many studies have explored ethnic variations in pathways to care, and it has been well documented that black Caribbean and black African patients are more likely than white patients to have pathways to care involving coercion and compulsory admissions (Bhui et al., 2003; Morgan et al., 2005a, 2005b). Similar findings have also been reported in other European countries (Lay et al., 2006; Norredam et al., 2010. The reasons for such differences remain poorly understood (Singh et al., 2007) and methodological limitations, such as very broad categorizations of ethnic group, small sample sizes and limited adjustment for potential confounding factors, have characterized much research in this area. Most previous work has not attempted to investigate differences in perceived reasons for admission and whether these might contribute to understanding differences in acute admission routes and pathways to care.

Debate still rages as to the extent of the part played by racism, institutional or individual, and stereotyping of black youth, especially male, in their high rates of psychotic diagnoses and vulnerability to coercion in the mental health system (McKenzie and Bhui, 2007; Singh, 2007). A review of the literature on the causes for the elevated rate of psychosis diagnoses among black Caribbeans living in the UK concludes that multiple risk factors are involved, with isolation and social exclusion (to which racism may contribute) playing a particularly important role (Pinto et al., 2008). Explanations in terms of clinician bias are limited by studies showing that the difference in incidence rates persists when diagnoses are made by ethnically matched or blinded raters (Fearon et al., 2006). It can still be argued, however, that the western term ‘psychosis’ is defined in a way that renders certain groups more likely to receive the diagnosis (Fernando, 1998).

Attention and debate has often focused especially on young black men and their adverse experiences of the mental health system, particularly following a number of high-profile public enquiries that have criticized their care. Evidence about black women’s experiences is less clear and consistent, and relatively few studies report findings disaggregated by gender for large samples of women, especially regarding pathways to care. However, there are some indications that gender may modify the association between ethnicity and compulsory admission (e.g. Bebbington et al., 1994; Morgan et al., 2005a), with black Caribbean men the most likely to be compulsorily admitted and large variations in the extent to which black women have been found to have excess rates of detention (Bebbington et al., 1994). Gender as well as ethnicity influences consultation rates in and referral rates from primary care and casualty departments (Lloyd and St Louis, 1996; Morgan et al., 2005a), so that clear evidence is needed as to the extent to which black women as well as men experience complex and coercive routes to care. Other minority ethnic groups have also been found to experience higher rates of compulsory detention (Ali et al., 2007; Morgan et al., 2005a) and again the evidence is limited as to how far this also applies to a range of female groups.

This study thus focuses solely on ethnic differences among women, examining rates of compulsory detention for a substantial sample in four London boroughs with very ethnically mixed populations. All four areas had women’s crisis houses as well as acute hospital wards. The crisis houses accepted only voluntary admissions. They were included in the samples in order to obtain a full overview of ethnic differences in response to the identified need for acute admission in the study catchment areas. To allow exploration of possible mediating factors that may contribute to explaining any differences found in rates of compulsory detention, clinician-rated reasons for admission and pathways to care were also investigated, including whether or not patients initiated help-seeking in the crisis. This exploration of potential mediators is a novel aspect of the investigation in a UK context. One similar investigation from the Netherlands is reported by Vinkers et al. (2010). They found that psychiatrists’ cited reasons for compulsory admission showed ethnic differences, with people from minority ethnic backgrounds more often reportedly admitted because of violence towards others. The authors argue that it was unclear how far this was a difference in presentations and how far a difference in psychiatrists’ perceptions.

### Aims

1. To investigate ethnic variations in compulsory and voluntary admissions among women.
2. To investigate, as potential mediating factors explaining any differences found in whether admissions are compulsory, (a) pathways to care and (b) clinician-rated reasons for admission.

### Main hypotheses

1. Women from black groups (black Caribbean, black African and black other) are more likely to be admitted under the Mental Health Act than white British women.
2. All groups of black women are more likely to have adverse pathways to care involving the criminal justice system than white British women.

This paper describes tests of these primary hypotheses, with adjustment for potentially confounding demographic and clinical variables. Further exploratory analyses aimed at identifying potential mediating factors (help-seeking and care pathway, clinician-rated reasons for admission) that may contribute to explaining any ethnic differences in the rates of compulsory detention will also be described.

A secondary aim throughout the analyses is to compare white other (including all white groups other than British) women with white British women, as so far little is known about their service use patterns, justifying exploratory analysis.

## Method

### Setting

The study took place in three inner London boroughs (Camden, Islington and Lambeth) and one borough of a more suburban character (Croydon). It formed one of the elements in the MRC-funded CHOICES study (Howard et al., in press), whose main aims have been the delineation of pathways to care to women’s crisis houses and to standard inpatient care, and the development of a method for comparing these forms of care.

### Sample

All women admitted both to standard acute wards and to crisis houses during a 12-week period in 2006 were included in this phase of the CHOICES study. For the analyses reported in this paper, only women from white British, white other, black Caribbean, black African and black other groups were included, as other groups were too small for analysis. Ethical approval was obtained from the University College London Hospital Research Ethics Committee.

### Instruments and data collection

Data were collected on sociodemographic characteristics and diagnosis, using the 2001 UK census categories to record ethnicity. Clinicians in the inpatient services and crisis houses were asked to record pathways to care, operationalized using questions from the WHO Pathways to Care Schedule (Gater et al., 1991). A modified version of the Reasons for Admission Questionnaire (Flannigan et al., 1994) was also included, with some additional reasons included following consultation with the study steering group and piloting. Additional information was sought from other clinicians involved with patients where full information was not available from acute wards, and the crisis and home treatment teams responsible for gate-keeping for acute admissions were contacted to corroborate and complete information on pathways to care for all patients. Finally, clinicians also rated the Threshold Assessment Grid (TAG, Slade et al., 2000). This is a standardized brief assessment of the severity of an individual’s mental health problems and associated risks; when rated by mental health professionals it has an intra-class correlation for total TAG scores of 0.58 (Slade et al., 2000).

Medical staff involved in patients’ care recorded their diagnoses, categorizing them as ‘schizophrenia or related disorder’ (schizophrenia, schizoaffective disorder, schizotypal disorder, other non-affective psychotic disorders), ‘affective disorder’ (depressive disorder, bipolar affective disorder) and ‘other diagnosis/no fixed diagnosis’ (mental and behavioural disorder due to drug/psychoactive substance use, personality disorder, conduct disorder, anxiety disorders, adjustment disorders and eating disorders).

### Analysis

Descriptive statistics and univariate tests were used to describe and explore baseline differences in social and clinical characteristics between ethnic groups. χ*^2^* tests were then used to explore whether ethnic group was associated first with compulsory admission and pathway to care involving the criminal justice system (the primary hypotheses), and second with admission to a crisis house and with other main features of the pathway to care (whether the patient sought help from mental health services herself in the crisis, whether family members sought help on her behalf, whether primary care staff were involved in seeking help and whether a community mental health team was involved). Where a significant association was found, logistic regression analysis was used to examine the odds for each ethnic group individually, adjusting for baseline social and clinical characteristics.

A similar procedure was used for an exploratory analysis of whether reasons for admission and TAG score appeared to be associated with ethnic group. Each potential reason for admission was rated as not a reason for admission, a minor reason or a major reason for each patient. Relatively few ratings of major reason for admission were made, and for the purposes of this analysis minor and major were grouped together yielding a dichotomous variable. The four TAG scores assessing risk could not be treated as interval level data as they deviated considerably from a normal distribution; they were therefore also transformed into dichotomous variables, with moderate, severe and very severe risk grouped together and compared with no or mild risk. Total TAG score was used as an indicator of severity and was approximately normally distributed.

A logistic regression analysis with data entered in blocks was then used to explore whether ethnic differences in rates of compulsory admission persisted when adjustment was made for reasons for admission and for pathways to care. First to be examined were the effects on the odds ratio between ethnic groups and compulsory admission of adding the reasons for admission variables; this showed reason for admission as a further explanatory variable in a model that already contained ethnic group and the main social and clinical characteristics. Second, pathways to care were added as a third block of variables in this model in order to explore whether differences in help-seeking could account for any of the differences found between ethnic groups in whether admission was compulsory. Last to be explored was whether the relationship between ethnic groups and risk of compulsory admission changed when TAG scores rather than reasons for admission were used as indicators of the nature and severity of clinical and social problems at admission.

Each of the baseline and pathways to care variables had some missing data, though none for more than 27 out of the 287 women. To avoid losing large numbers of cases in the regression analyses below, for most of the baseline variables the assumption was made that a missing value meant that the attribute concerned was not present (e.g. people with missing data on employment were assumed not to be working for the purposes of the multiple regressions; those with missing data for whether they had children at home were assumed not to have). To test whether making this assumption might have introduced some form of bias, as a sensitivity analysis the analyses were concluded by repeating the main regressions with the opposite assumption regarding missing data (e.g. those with missing data for employment were assumed to be working). This resulted in no substantial change in the main findings and is therefore not reported further.

## Results

### Sample characteristics

During the three-month period of the study, 339 women were admitted to a psychiatric inpatient ward or crisis house (77% to hospital; 23% to a crisis house). Admission procedures involved recording self-ascribed ethnic group and some importance was attached to the completeness of this information (it was used as a performance indicator within the services), but data on ethnic group were unavailable for six. A further 46 women belonged to ethnic groups represented only in very small numbers in this sample and were therefore excluded as comparisons involving them would have lacked power.

This yielded a total sample of 287 for the analyses reported in this paper, classified as white British, white other (including here the white Irish census category), black Caribbean, black African, and black other. The black other group were predominantly women who described themselves as black British. The mean age of the sample was 40.1 years (*SD* 12.1, range 18–69). There were no significant demographic differences between ethnic groups (Table 1).

**Table 1.** Sociodemographic and clinical characteristics of white UK, white other, black Caribbean, black African and black other women admitted to acute mental health services

Variable (*n* =)^1^	Ethnic background					*p*^2^
	White British (*n* = 146)	White other (*n* = 45)	Black Caribbean (*n* = 26)	Black African (*n* = 41)	Black other (*n* = 29)	
**Age: mean** (*SD*)	40.34 (12.8)	44.11 (11.0)	41.46 (12.4)	37.4 (11.5)	36.3 (9.9)	0.15
**Employed**						
Yes	16 (11.7)	2 (5)	3 (11.5)	7 (17.9)	4 (16)	0.47
No	121 (88.3)	38 (95)	23 (88.5)	32 (82.1)	21 (84)	
**Children under 16**						
Yes	34 (25.6)	8 (20)	5 (20)	17 (42.5)	14 (51.9)	0.11
No	99 (74.4)	32 (80)	20 (80)	23 (57.5)	13 (48.1)	
**Living alone before the admission**						
Yes	71 (50)	16 (42.1)	16 (64)	19 (48.7)	6 (25)	0.78
No	71 (50)	22 (57.9)	9 (36)	20 (51.3)	18 (75)	
**Supported accommodation**						
Yes	10 (7.3)	6 (15)	3 (11.5)	5 (12.8)	2 (7.1)	0.57
No	127 (92.7)	34 (85)	23 (88.5)	34 (87.2)	26 (92.9)	
**Previous psychiatric admissions**						
Yes	115 (81.5)	31 (73.9)	18 (75)	32 (82.1)	22 (78.9)	0.80
No	26 (18.5)	11 (26.1)	6 (25)	7 (17.9)	6 (21.1)	
**Previous contact with mental health services**						
Yes	116 (83.5)	32 (71.4)	24 (92.3)	34 (85)	23 (79.3)	0.28
No	23 (16.5)	12 (28.6)	2 (7.7)	6 (15)	6 (20.7)	
**Primary diagnosis at time of admission**						
Affective disorder	56 (43.1)	17 (43.6)	6 (25)	12 (30)	7 (25.9)	< 0.005
Schizophrenia or related disorder	37 (28.5)	12 (30.8)	17 (70.8)	24 (60)	14 (51.9)	
Other disorder /no fixed diagnosis	37 (28.5)	10 (25.6)	1 (4.2)	4 (10)	6 (22.2)	

1 Variables had a different number of missing cases, ranging from two (age) to 27 (clinical diagnosis)

2 The *p* values are derived from significance tests for the association between each of the variables and ethnic group, using analysis of variance for age and χ^2^ for the rest

3 Numbers and percentages in each group are shown for all the categorical variables

### Psychiatric diagnosis

Rates of schizophrenia-related diagnoses were lowest among the white groups of patients and highest among black African women (Table 1). Black African and black Caribbean women had the lowest rates of other (not affective and not schizophrenia-related) disorders; the main sub-group in this category were people with personality disorders.

### Where admitted

Ethnic group was significantly associated overall with whether patients were admitted to hospital or to a crisis house (Table 2). White British women were more likely than any other group to be admitted to a crisis house, although the difference did not quite reach statistical significance for the white other group. The black groups were combined for this analysis only as the fact that no black other women were admitted to the crisis house prevented computation of individual odds ratios for each group. Overall the odds of admission to the crisis house were almost four times greater for white British women as for women in one of the black groups.

**Table 2.** Type of admission and pathway to care

Variable (*n* =)^1^	Ethnic background					*p*
	White British(*n* = 146)	White other(*n* = 45)	Black Caribbean (*n* = 26)	Black African(*n* = 41)	Black other(*n* = 29)	
**Nature of admission Where admitted**						
Ward	100 (68.5)	37 (82.2)	22 (84.6)	36 (87.8)	29 (100)	0.01
Crisis house	46 (31.5)	8 (17.8)	4 (15.4)	5 (12.2)	0 (0)	
Adjusted *OR^2^* for crisis house	1.0	0.44	All black groups pooled: 0.26			
(95% *CI*)		(0.18–1.04)		(0.12–0.60)		
*P*		**0.06**		**0.001**		
**Mental Health Act status at admission**						
Voluntary	125 (86.8)	29 (64.4)	15 (57.7)	21 (51.2)	16 (55.2)	< 0.0005
Detained	19 (13.2)	16 (35.6)	11 (42.3)	20 (48.8)	13 (44.8)	
Adjusted *OR* for compulsory admission	1.0	3.53	3.88	5.80	5.22	
(95% *CI*)		(1.57–7.94)	(1.47–10.2)	(2.52–13.4)	(2.06–13.2)	
*P*		**0.002**	**0.006**	< **0.0005**	**0.001**	
**Pathway to care**						
**Police or criminal justice system in pathway**	25 (17.1)	15 (33.3)	8 (30.1)	17 (41.5)	14 (48.3)	< 0.0005
Adjusted	1.0	2.81	2.18	3.33	4.27	
*OR* (95% *CI*)		(1.27–6.21)	(0.80–5.93)	(1.47–7.53)	(1.74–10.5)	
*P*		**0.01**	**0.13**	**0.004**	**0.002**	
**A&E attendance in pathway**	80 (54.8)	20 (44.4)	14 (53.9)	15 (36.6)	17 (58.6)	0.27
**Primary care involvement in pathway**	23 (15.8)	6 (13.3)	1 (4.0)	4 (9.8)	1 (3.4)	0.22
**CMHT involvement in pathway**	90 (61.6)	31 (68.9)	14 (53.8)	29 (70.7)	15 (51.7)	0.37
**Self-referral to mental health services for help with crisis**	73 (52.9)	10 (27.8)	6 (24.0)	8 ( 20.5)	5 (17.9)	< 0.0005
Adjusted *OR*	1.0	0.33	0.36	0.28	0.23	
(95% *CI*)		(0.14–0.76)	(0.13–1.01)	(0.12–0.69)	(0.08–0.66)	
*P*		**0.009**	**0.052**	**0.005**	**0.007**	
**Family involved in pathway to admission**	24 (16.4)	7 (15.6)	2 (7.7)	14 (34.1)	9 (31.0)	0.02
Adjusted *OR*	1.0	0.83	0.42	2.56	1.55	
(95% *CI*)		(0.32–2.19)	(0.87–2.08)	(1.04–6.04)	(0.59–4.06)	
*P*		**0.71**	**0.29**	**0.04**	**0.37**	

1 Different numbers of cases are missing for each variable (up to a maximum of 21 for Self-referral to mental health services for help with crisis)

2 Adjusted odds ratios appear for those variables where an initial logistic regression by ethnic category indicated a significant difference between groups. The potentially confounding background variables for which adjustment has been made are age, whether currently employed, whether in supported accommodation, whether living alone, whether already in contact with mental health services before crisis, whether any history of past admissions, whether has any children and diagnosis (grouped as schizophrenia and other non-affective psychosis, affective disorders and other or no diagnosis)

### Rates of compulsory detention

In total, 27.1% (*n* = 79) of patients were compulsorily admitted under the UK Mental Health Act (MHA). These represented 34.6% of all hospital admissions, but no crisis house admissions as these are all voluntary. Ethnic background was significantly associated with being detained as hypothesized (*p* < 0.005), with significantly higher odds of being compulsorily admitted not only for each of the black groups but also for the white other group. Overall, 13.2% of white British women were admitted compulsorily, compared to 35.6% of white other, 42.3% of black Caribbean, 48.8% of black African and 44.8% of black other women (Table 2). These differences remained statistically significant after adjusting for social and clinical characteristics, including diagnostic group, which was not significantly associated with compulsory admission in this model.

The same pattern was found when hospital admissions were considered alone, and all differences remained highly significant (Table 3).

**Table 3.** Hospital group only: Variables associated with ethnic group

Variable (*n* =)^1^	Ethnic background					*p*
	White British (*n* = 100)	White other (*n* = 37)	Black Caribbean (*n* = 20)	Black African (*n* = 22)	Black other (*n* = 29)	
**Nature of admission Mental Health Act status at admission**						
Voluntary	79 (80.6)	21 (56.8)	11 (50.0)	16 (44.4)	16 (55.2)	< 0.0005
Detained	19 (19.4)	16 (43.2)	11 (50)	20 (55.6)	13 (44.8)	
Adjusted *OR* for compulsory admission	1.0	3.26	4.03	5.78	3.76	
(95% *CI*)		(1.38–7.70)	(1.43–11.37)	(2.34–14.3)	(1.44–9.81)	
*P*		**0.007**	**0.009**	< **0.0005**	**0.007**	
**Pathway to care Police or criminal justice system in pathway**	21 (21.0)	14 (37.8)	8 (36.4)	17 (47.2)	14 (48.3)	0.010
Adjusted *OR*	1.0	2.56	2.23	3.43	3.49	
(95% *CI*)		(1.08–6.08)	(0.77–6.45)	(1.41–8.37)	(1.36–8.92)	
*P*		**0.01**	**0.14**	**0.007**	**0.009**	
**Self-referral to mental health services for help with crisis**	50 (53.2)	6 (21.4)	5 (23.8)	7 (20.0)	5 (17.9)	< 0.0005
Adjusted *OR*	1.0	0.23	0.38	0.27	0.22	
(95% *CI*)		(0.08–0.65)	(0.12–1.22)	(0.10–0.72)	(0.07–0.68)	
*P*		**0.005**	**0.10**	**0.009**	**0.008**	
**Family involved in pathway to admission**	15 (15.0)	5 (13.5)	2 (9.1)	14 (38.9)	9 (31.0)	0.006
Adjusted *OR*	1.0	0.73	0.51	3.34	1.52	
(95% *CI*)		(0.23–2.34)	(0.09–2.72)	(1.21–9.34)	(0.54–4.34)	
*P*		**0.60**	**0.43**	**0.02**	**1.52**	

### Pathways to care

There were some notable differences between ethnic groups in their pathways to inpatient admission (Table 2). Overall, there was a highly significant association between ethnic group and an adverse pathway to care, defined here as police or criminal justice system involvement at some point in the pathway to admission. Looking individually at the ethnic groups in the study, women from white other, black African and black other groups were more likely to reach services by such a route; the difference for the black Caribbean group did not quite reach statistical significance. Conversely, white British women were significantly more likely than all other groups except black Caribbeans to have initiated help-seeking themselves. Family involvement in help-seeking was also significantly associated with ethnic group, but the only individual group found to be significantly different from the white British groups were black Africans, with family involved in seeking help for 34% compared to 16% of white British patients. No significant association was found between ethnic group and attendance in the accident and emergency department (the UK equivalent of the emergency room) in the course of the pathway to care, or in community mental health team involvement and primary care involvement; the latter was at a low level across all groups.

Very similar patterns were observed when hospital admissions only were analyzed (Table 3).

### Reasons for admission

There were also a number of ethnic differences in clinicians’ ratings of reasons for admission (Table 4). White British women were more likely than black women from any background to be reported to have been admitted because of risk of suicide, and less likely than any other group to have been admitted because of an assault on someone outside the family or destruction of property. Relapse due to not taking medication or need for treatment of a physical problem were more likely to be rated as contributing to admission for white other women than for white British women, although these findings were only of marginal significance and it should be noted that adjustment has not been made for multiple testing in these exploratory analyses.

**Table 4.** Reasons for admission by ethnic group: Factors identified by clinicians as major or minor contributors

Variable	Ethnic background					*p*
	White British (*n* = 146)	White other (*n* = 45)	Black Caribbean (*n* = 26)	Black African (*n* = 41)	Black other (*n* = 29)	
**Supervision required**	116 (85.3)	34 (56.7)	20 (76.9)	34 (89.5)	28 (100)	0.14
**Intensive observation/assessment**	101 (73.7)	31 (79.5)	20 (74.1)	32 (82.1)	26 (92.9)	0.24
**Risk of suicide**	84 (60.9)	21 (52.5)	8 (30.1)	12 (31.6)	8 (28.6)	< 0.0005***
Adjusted *OR*	1.0	0.73	0.36	0.36	0.26	
(95% *CI*)		(0.35–1.53)	(0.14–0.93)	(0.16–0.80)	(0.10–0.67)	
*P*		**0.40**	**0.035**	**0.012**	**0.005**	
**Risk of self-neglect**	79 (57.7)	79 (57.7)	26 (63.4)	19 (73.1)	16 (55.2)	0.426
**Assault on patient by other(s)**	16 (11.7)	5 (12.5)	4 (15.4)	1 (2.6)	2 (7.4)	0.393
**Physical health deterioration**	47 (34.1)	16 (40)	11 (42.3)	11 (28.2)	6 (20.7)	0.384
**Sexually inappropriate behaviour**	14 (10.1)	7 (16.7)	2 (7.7)	9 (23.7)	1 (3.4)	0.071
**Assault on related children**	4 (2.9)	1 (2.5)	3 (11.5)	3 (7.9)	1 (3.4)	0.252
**Assault on relative(s)**	8 (5.8)	2 (5)	2 (7.6)	3 (7.9)	4 (13.8)	0.614
**Assault on other(s) – not family**	6 (4.3)	9 (22)	9 (38.5)	7 (17.9)	4 (13.8)	< 0.0005***
Adjusted *OR*	1.0	6.55	9.15	3.9	5.52	
(95% *CI*)		(2.03–21.1)	(2.69–31.1)	(1.13–13.2)	(1.54–19.8)	
*P*		**0.002**	<**0.0005**	**0.03**	**0.009**	
**Destruction of property**	8 (5.8)	7 (17.5)	5 (19.2)	8 (21.1)	6 (20.7)	0.019*
Adjusted *OR*	1.0	3.90	3.43	4.06	4.07	
(95% *CI*)		(1.28–11.9)	(1.33–12.4)	(1.33–12.4)	(1.23–13.5)	
*P*		**0.017**	**0.014**	**0.014**	**0.022**	
**Relapse / not taking medication**	44 (32.4)	20 (48.8)	16 (61.5)	22 (56.4)	14 (48.3)	0.008*
Adjusted *OR*	1.0	2.55	2.27	2.24	2.26	
(95% *CI*)		(1.14–5.70)	(0.87–5.92)	(0.98–5.13)	(0.90–5.67)	
*P*		**0.022**	**0.092**	**0.056**	**0.083**	
**Misuse of medication or drugs**	48 (35.3)	16 (39)	8 (30.8)	5 (13.2)	8 (27.6)	0.091
**Treatment of physical problem needed**	17 (12.2)	12 (30.8)	5 (19.2)	2 (5.3)	3 (7.7)	0.022*
Adjusted *OR*	1.0	2.66	1.89	0.37	0.98	
(95% *CI*)		(1.07–6.64)	(0.56–6.37)	(0.08–1.82)	(0.25–3.85)	
*P*		**0.036**	**0.30**	**0.22**	**0.97**	
**Care of personal hygiene**	27 (19.6)	11 (27.5)	9 (34.6)	11 (28.9)	2 (6.9)	0.076
**Admission at client’s request**	55 (40.4)	10 (25)	4 (15.4)	8 (21.1)	7 (33.3)	0.022*
Adjusted *OR*	1.0	0.53	0.32	0.40	0.48	
(95% *CI*)		(0.23–1.21)	(0.10–1.02)	(0.16–1.002)	(0.18–1.13)	
*P*		**0.13**	**0.055**	**0.051**	**0.14**	
**Removal from harm/stressful situation**	63 (46)	24 (58.5)	9 (34.6)	11 (28.9)	7 (31.9)	0.072
**Relief of carers/relatives**	26 (7.2)	9 (23)	5 (25)	11 (28.9)	7 (31.9)	0.74
**Admission at family’s request**	31 (22.5)	6 (15)	2 (8)	12 (31.6)	7 (31.9)	0.19

* *p* < 0.05, ** *p* < 0.005, *** *p* < 0.0005

### Ethnic variations in severity of risks and problems

The possibility that there are differences in the perceived problems triggering admission was also explored using TAG ratings of risks and total score (Table 5). No differences were found between ethnic groups in overall severity, risk of unintentional self-harm or risk from others, but white British women were again perceived as at higher risk of self harm than the black groups, and risk to others also showed an association with ethnicity, though only the black other group had a clearly elevated perceived risk to others compared with the white British group.

**Table 5.** Threshold Assessment Grid scores by ethnic group

TAG variable (coded as binary)^1^	Ethnic background					*p*
	White British (*n* = 146)	White other (*n* = 45)	Black Caribbean (*n* = 26)	Black African (*n* = 41)	Black other (*n* = 29)	
**Risk of intentional self-harm**	73 (54.5)	15 (38.5)	3 (13.0)	6 (15.8)	7 (24.1)	< 0.0005
	1.0	0.55	0.17	0.20	0.26	
		(0.25–1.19)	(0.05–0.65)	(0.07–0.53)	(0.10–0.68)	
		**0.13**	**0.0009**	**0.001**	**0.006**	
**Risk of unintentional self-harm**	61 (45.5)	18 (46.2)	8 (33.3)	16 (42.1)	15 (53.6)	0.68
**Risk from others**	32 (23.9)	15 (36.6)	5 (20.8)	6 (15.8)	7 (24.1)	0.29
**Risk to others**	25 (18.7)	13 (32.5)	9 (37.5)	13 (34.2)	15 (51.7)	0.003
	1.0	2.29	1.63	1.67	4.51	
		1.0–5.29	0.60–4.41	(0.67–3.98)	1.81–11.2	
		**0.052**	**0.34**	**0.25**	**0.001**	
**Mean total TAG score for each group**	8.9	9.1	7.8	7.5	8.8	0.16

1 As their distribution was very deviant from normal, TAG variables related to risk were recoded into binary variables: no or mild risk in one category; moderate, severe or severe risk in the other

### The effects on ethnic differences of adjusting for reasons for admission and pathways to care

The final stage of the analysis involved the exploration of whether adjusting for reasons for admission and then for pathways to care influenced the ethnic differences found in compulsory admission (Table 6). As previously described, when ethnic group and the main social and clinical characteristics variables were entered into a regression, all other groups were significantly more likely to be detained than the white British group. Past history of admission was associated with a greater likelihood of admission being voluntary; no other variable entered at this stage was significantly associated with risk of compulsory detention.

**Table 6.** Models of factors associated with compulsory admission among women

Ethnic group variables and other variables significant at *p* = 0.05 level in the model	*OR* (95% *CI*)	*p*
**Version 1. Social and clinical characteristics, including ethnicity entered at Step 1**		
White UK	1.0	
White other	3.53 (1.57–7.94)	0.002**
Black Caribbean	3.88 (1.47–10.2)	0.006*
Black African	5.80 (2.52–13.4)	< 0.0005***
Black other	5.22 (2.06–13.2)	0.001**
Past history of admission	0.41 (0.19–1.85)	0.017*
Percentage correctly classified by model: 76%χ*^2^* = 42.3, *df* = 13, *p* < 0.0005		
**Version 2. Step 1 variables as for Version 1, reasons for admission that showed significant adjusted ethnic differences (see Table 4) entered on Step 2**		
White UK	1.0	
White other	2.95 (1.24–7.03)	0.015*
Black Caribbean	2.37 (0.81–6.89)	0.11
Black African	4.02 (0.65–9.82)	0.002**
Black other	3.22 (1.17–8.86)	0.024*
Past history of admission	0.40 (0.18–0.86)	0.02*
Suicide risk	0.27 (0.14–0.54)	< 0.0005***
Assault on others (not family)	2.53 (1.05–6.07)	0.038*
Percentage correctly classified by model: 81%χ*^2^* = 69.7, *df* = 18, *p* < 0.0005		
**Version 3. Steps 1 and 2 as for Version 2, pathways to care variables that showed significant adjusted ethnic differences (see Table 2) entered on Step 3**		
White UK	1.0	
White other	1.98 (0.76–5.13)	0.16
Black Caribbean	2.0 (0.64–6.27)	0.24
Black African	3.11 (1.18–8.18)	0.021*
Black other	2.21 (0.76–6.43)	0.14
Past history of admission	0.39 (0.17–0.91)	0.029*
Suicide risk	0.43 (0.20–0.91)	0.027*
Self referral to mental health services for help with crisis	0.32 (0.13–0.78)	0.012*
Police or criminal justice system involved in pathway	4.12 (2.07–8.19)	< 0.0005***
Percentage correctly classified by model: 82%χ*^2^* = 96.4, *df* = 21, *p* < 0.0005		

* *p* < 0.05, ** *p* < 0.005, *** *p* < 0.0005

The reasons for admission variables found to be associated with ethnic group were then added to the model. Suicide risk (which increased the likelihood of voluntary admission) and assault on non-family members (which increased the likelihood of compulsory admission) were significant in this model. Adding these variables reduced all the odds ratios for ethnic groups with compulsory admission and reduced their significance levels, and the black Caribbean group no longer had significantly greater odds of compulsory admission with this adjustment.

Pathways to care variables were then also added. Police or criminal justice system involvement was associated with a four-fold increase in the odds of compulsory admission, while those who had sought help from mental health services themselves had substantially lower odds of compulsory admission. When these variables were included in the model, the odds ratios for ethnic groups with compulsory admission were further reduced and only black African ethnicity was still associated with greater risk of compulsory admission at a statistically significant level.

The same procedure was followed substituting TAG variables for the reasons for admission variables. This resulted in a closely similar model but with the TAG ‘deilberate self-harm’ variable rather than self-harm as a reason for admission included in the model alongside being black African, self-referral to mental health services, and police or criminal justice system involvement in the pathway to care.

## Discussion

The principal findings of this study were, as hypothesized, that black women were more likely than white British women to be compulsorily rather than voluntarily admitted to hospital, and black African and black other (though not black Caribbean) women were also more likely to have police or criminal justice system involvement in their route to care. Similar patterns were found for white patients not from British backgrounds. White British women were more likely than all other groups to be admitted to a crisis house rather than to hospital.

Differences also emerged in reasons for admission and pathways to care. White British women were more likely than others actively to seek help, and suicidal risk was more likely to be the clinician-perceived reason for admission. According to clinicians, white other and black women were more likely to be admitted because of an assault on an unrelated person or destruction of property. Adjusting for differences in reasons for admission and pathways to care reduced the ethnic differences in compulsory admission, raising the possibility that ethnic differences in the triggers to admission or routes to care are potential explanatory or mediating factors in the large differences found in rates of compulsory admission.

### Rates of compulsory admission for women

The findings for black women correspond to most of the previous studies investigating compulsory admissions in black patients in general: the odds ratios obtained are close to the pooled odds ratio of 4.48 reported for the odds of being detained under a civil section for black compared to white patients from a meta-analysis by Singh and colleagues (2007). Comparing the present findings with this meta-analysis suggests that differences in rates of compulsory admission are as great for black women as for men, weakening arguments that specific discrimination against and negative stereotypes of young black men might be a cause of excess compulsory admissions.

A previously unreported finding is the excess of compulsory detention among the white other group. This suggests that it is inappropriate to group together white British and other white groups when epidemiological comparisons are made. It also suggests a need to investigate this group’s experiences of mental health services, especially as many recent migrants to the UK and other affluent countries, for example economic migrants from Eastern Europe and refugees and asylum seekers from former Yugoslavia, the Middle East and North Africa, fall within this group. The service use patterns shown by white others in this study are considerably closer to those of the black groups than to the white British population on most of the variables examined, suggesting that the explanation for differences should be sought in the general experience of being a migrant or descendant of migrants rather than in membership of a specific racial or cultural group.

### Reasons for admission and ethnic differences in compulsory admission

The reasons for high rates of compulsory admission have been extensively debated (Singh et al., 2007). Possibilities include lower satisfaction with mental health services (Parkman et al., 1997) (although a recent large-scale analysis (Raleigh et al., 2007) did not find such differences), expectations among black communities of racist mistreatment by mental health services (McLean et al., 2003), institutional racism in psychiatric services (McKenzie and Bhui, 2007), diagnostic differences or presentations of mental illness characterized by more severe disturbance (Bebbington et al., 1994), stereotyping of black people as violent (Webber and Huxley, 2004) and an under-utilization of GPs for mental health reasons (Burnett et al., 1999; Cole et al., 1995). Aversive relationships with services have been hypothesized to develop over time. For example, Burnett et al. (1999) found that it was the readmissions rather than the initial admissions of black Caribbean patients that were more likely to have been compulsory.

Relatively little empirical evidence is available to allow direct evaluation of the above possibilities (Singh et al., 2007). Against this background, the present findings regarding the effects of adjusting for social and clinical variables, clinician-rated reasons for admission and severity of risks and disturbance, and pathways to care may provide useful starting points for further investigations. No evidence was found to support explanations in terms of diagnosis or severity of disturbance. Ratings regarding self-harm and violence were related to risk of compulsory admission, and adjusting for these somewhat reduced the strength of association between ethnic group and compulsory admission, though it was still present for most of the groups. As all the information came from clinicians, it is not possible to discern whether these apparent differences regarding risks of self-harm and assault are a result of stereotyping by clinicians or whether they reflect real differences between ethnic groups in how they present when ill.

International research suggests that suicide rates are higher among white than black populations (Garlow et al. 2005; McKenzie et al., 2003), with one US study finding that white females were three times more likely to commit suicide than black females (Runyan et al., 2003). Crawford et al. (2005) found that lifetime suicidal ideation was lower among ethnic minorities (with the exception of white Irish men) than among white British people. A more complex picture emerges using a narrower ethnic classification, and with the inclusion of other social and demographic variables. High suicide rates have been observed in Indian and East-African born women (Bhui and McKenzie, 2008; Bhui et al., 2007) and the lower rates previously documented in Caribbean men may be specific to older generations (McKenzie et al., 2003). Thus, differences in risk ratings found in the present study may reflect some real ethnic differences in suicidality. Understanding this further is again a question for future research, but the present results suggest that an actual or perceived difference in behaviour when unwell might contribute to explaining ethnic differences.

### Pathways to admission

High rates of adverse pathways to care among black patients are well documented (Bhui et al., 2003). In the present sample, the rates of police and criminal justice system contact in the pathway to care for the black Caribbean group were raised, but not significantly so, confirming a need to disaggregate black groups. However, being black African or black other was strongly associated with such a route to services. The replication of this pattern in a female-only sample suggests that it cannot solely be a result of a troubled relationship between young black men and the police, especially as it was also found for the white other group.

Previous research has found that black patients are less likely to make voluntary contact with services (e.g. McGovern and Cope, 1991) and they are less likely to see a GP before an admission (e.g. Morgan et al., 2005b). The present results are partly consistent with these findings: all other groups were markedly less likely than white British patients to have self-referred for help from mental health services in their current crisis. However, no significant ethnic differences were found in this study in primary care involvement, which was low across all ethnic groups. The latter finding should be interpreted with caution as there was limited information available about whether primary care was involved.

When adjustment is made for these differences in pathways to care, the ethnic differences in whether admission is compulsory are considerably reduced, suggesting they are relevant to understanding the excess of compulsory admission. Lower rates of self-referral in the crisis and higher rates of police involvement may well signal important differences in attitudes to and expectations of mental healthcare. They may also indicate that there are ethnic differences in the response patients get when they try to seek help. The finding that black Africans have both high rates of police involvement in admission and higher rates of family involvement in pathway to admission may reflect Morgan et al.’s (2005b) observation that family and friends appear to initiate help-seeking via the police more often for black service users than for others.

### Understanding differences in rates of compulsory admission

Returning to the potential explanations for excess rates of compulsory detentions (Singh et al., 2007), the present findings do not support explanations in terms of more severe disturbance differences in primary care involvement or diagnostic differences. Given the wide range of groups involved, they also suggest that if a cultural explanation is invoked, it needs to be one that is applicable to rather a broad range of groups.

Differences in pathway to care seemed to have the most explanatory value in the study’s models when accounting for the differences in compulsory admission. This suggests that further attempts to understand excess rates should involve careful examination of the help-seeking patterns of immigrant groups compared to the indigenous population, of the experiences and views of mental health services and attitudes to mental illness that may underlie these, and of whether there are differences by ethnic group in the responses people get when they seek help for themselves or for their relatives. Further research is then needed about how to improve service access for black and other minority ethnic people. Two recent systematic reviews of the published and grey literature conclude that while some innovations show promise, good-quality evaluative research in this area is lacking (Moffat et al., 2009; Sass et al., 2009). Studies seeking the views of ethnic minority service users themselves will be useful here, and are relatively scarce. Bowl (2007) carried out interviews and focus groups with South Asian service users, who identified socioeconomic, cultural and institutional exclusion as impacting on their experience of mental health services.

### Crisis house admissions

Ethnic differences were also found in whether admission was to a crisis house or hospital. These ethnic inequalities in crisis house use may reflect white British women being more willing help-seekers or apparently less likely to be admitted because of an assault. However, it is also possible that the facilities are not perceived as being as accessible and appropriate by other ethnic groups, even though they are undoubtedly committed to serving a diverse local community and employ staff from a range of backgrounds. Being able to self-refer, though desirable in some ways, may also create unintended inequalities in use of crisis houses.

### Limitations

The results of this study should be considered in relation to its limitations. First, the categorization used in the study, although an improvement over a simple classification as white or black, still involves very heterogeneous groupings. The black African group, for example, includes people originating from countries many thousands of miles apart with vast differences in history and culture, while the white other group also encompasses great diversity, including people from Western and Eastern Europe, the Middle East, North Africa and both the Americas. Ethnic group was based on clinician recordings, which was inevitable given the method and had the benefit of resulting in complete data for this sample, but was contrary to the recommendation that ethnic group be self-ascribed. Local health service policy required staff to record patients’ self-ascribed ethnic group, but it was not possible to monitor how far this happened in practice. All data were collected from clinicians rather than the women themselves; this is likely to result in some inaccuracies and may also introduce bias, for example, due to racial stereotypes influencing ratings in areas such as violence. Although larger than a substantial number of previously investigated samples (Singh et al., 2007), numbers in each of the main ethnic groups were relatively small. Much of the analysis was exploratory rather than hypothesis-driven and no adjustment has been made for multiple testing, indicating a need for caution especially in relation to findings that are close to the conventional level for statistical significance.

The cross-sectional study design and retrospective nature of the ratings made by staff are further limitations to be considered in interpreting these models, which were limited to simple regression analyses without investigation of interactions or other more complex forms of relationship between variables. Finally, the four London boroughs from which information was collected are not typical of the UK population as a whole, though they do have the advantage for the current investigation of very high levels of ethnic diversity.

## References

[bibr1-0020764010382369] Ali S. Dearman S.P. McWilliam C. (2007) Are Asians at Greater Risk of Compulsory Psychiatric Admission than Caucasians in the Acute General Adult Setting? Medicine, Science and the Law 47(4), 311–314 10.1258/rsmmsl.47.4.311 18069536

[bibr2-0020764010382369] Bebbington P.E. Feeney S.T. Flannigan C.B. Glover G.R. Lewis S.W. Wing J.K. (1994) Inner London Collaborative Audit of Admissions in Two Health Districts. II: Ethnicity and Use of the Mental Health Act. British Journal of Psychiatry 165(6), 743–749 7881776 10.1192/bjp.165.6.743

[bibr3-0020764010382369] Bhui K. McKenzie K. Rasul F. (2007) Rates, Risk Factors and Methods of Self-Harm among Minority Ethnic Groups in the UK: A Systematic Review. BMC Public Health 7, 336 18021438 10.1186/1471-2458-7-336 PMC2211312

[bibr4-0020764010382369] Bhui K. Stansfeld S. Hull S. Priebe S. Mole F. Feder G. (2003) Ethnic Variations in Pathways to and Use of Specialist Mental Health Services in the UK: Systematic Review. British Journal of Psychiatry 182, 105–116 12562737 10.1192/bjp.182.2.105

[bibr5-0020764010382369] Bhui K.S. McKenzie K. (2008) Rates and Risk Factors by Ethnic Group for Suicides within a Year of Contact with Mental Health Services in England and Wales. Psychiatric Services 59(4), 414–420 18378841 10.1176/ps.2008.59.4.414

[bibr6-0020764010382369] Bowl R. (2007) The Need for Change in UK Mental Health Services: South Asian Service Users’ Views. Ethnicity & Health 12(1), 1–19 17132582 10.1080/13557850601002239

[bibr7-0020764010382369] Burnett R. Mallett R. Bhugra D. Hutchinson G. Der G. Leff J. (1999) The First Contact of Patients with Schizophrenia with Psychiatric Services: Social Factors and Pathways to Care in a Multiethnic Population. Psychological Medicine 29(2), 475–483 10218939 10.1017/s0033291798008125

[bibr8-0020764010382369] Cole E. Leavey G. King M. Johnson-Sabine E. Hoar A. (1995) Pathways to Care for Patients with a First Episode of Psychosis. A Comparison of Ethnic Groups. British Journal of Psychiatry 167(6), 770–776 8829745 10.1192/bjp.167.6.770

[bibr9-0020764010382369] Crawford M.J. Nur U. McKenzie K. Tyrer P. (2005) Suicidal Ideation and Suicide Attempts among Ethnic Minority Groups in England: Results of a National Household Survey. Psychological Medicine 35(9), 1369–1377 16168159 10.1017/S0033291705005556

[bibr10-0020764010382369] Fearon P. Kirkbride J.B. Morgan C. Dazzan P. Morgan K. Lloyd T. Hutchinson G. Tarrant J. Fung W.L. Holloway J. Mallett R. Harrison G. Leff J. Jones P.B. Murray R.M.; AESOP Study Group (2006) Incidence of Schizophrenia and other Psychoses in Ethnic Minority Groups: Results from the MRC AESOP Study. Psychological Medicine 36(11), 1541–1550 16938150 10.1017/S0033291706008774

[bibr11-0020764010382369] Fernando S. (1998) Race and Culture in Psychiatry. London: Routledge 10.1017/s0033291797226498 9572107

[bibr12-0020764010382369] Flannigan C.B. Glover G.R. Wing J.K. Lewis S.W. Bebbington P.E. Feeney S.T. (1994) Inner London Collaborative Audit of Admission In Two Health Districts. III: Reasons for Acute Admission to Psychiatric Wards. British Journal of Psychiatry 165(6), 750–759 7881777 10.1192/bjp.165.6.750

[bibr13-0020764010382369] Garlow S.J. Purselle D. Heninger M. (2005) Ethnic Differences in Patterns of Suicide across the Life Cycle. American Journal of Psychiatry 162(2), 319–323 15677597 10.1176/appi.ajp.162.2.319

[bibr14-0020764010382369] Gater R. de Almeida e Sousa B. Barrientos G. Caraveo J. Chandrashekar C.R. Dhadphale M. Goldberg D. al Kathiri A.H. Mubbashar M. Silhan K. (1991) The Pathways to Psychiatric Care: A Cross-Cultural Study. Psychological Medicine 21(3), 761–774 1946864 10.1017/s003329170002239x

[bibr15-0020764010382369] Howard L.M. Rigon E. Cole L. Lawlor C. Johnson S. (2008) Admission to Women’s Crisis Houses or to Psychiatric Wards: Women’s Pathways to Admission. Psychiatric Services 59(12), 1443–1449 19033172 10.1176/appi.ps.59.12.1443 PMC2615223

[bibr16-0020764010382369] Lay B. Lauber C. Nordt C. Rossler W. (2006) Patterns of Inpatient Care for Immigrants in Switzerland: A Case Control Study. Social Psychiatry and Psychiatric Epidemiology 41(3), 199–207 16424971 10.1007/s00127-005-0014-2

[bibr17-0020764010382369] Lloyd K. St Louis L. (1996) Common Mental Disorders among Africans and Caribbeans. In Bhugra D. Bhal V. (eds) Ethnicity: An Agenda for Mental Health. London: Gaskell

[bibr18-0020764010382369] McGovern D. Cope R. (1991) Second Generation Afro-Caribbeans and Young Whites with a First Admission Diagnosis of Schizophrenia. Social Psychiatry and Psychiatric Epidemiology 26(2), 95–99 2047912 10.1007/BF00791535

[bibr19-0020764010382369] McKenzie K. Bhui K. (2007) Institutional Racism in Psychiatry. Psychiatric Bulletin 31, 397

[bibr20-0020764010382369] McKenzie K. van Os J. Samele C. van Horn E. Tattan T. Murray R.; UK700 Group (2003) Suicide and Attempted Suicide among People of Caribbean Origin with Psychosis Living in the UK. British Journal of Psychiatry 183, 40–44 12835242 10.1192/bjp.183.1.40

[bibr21-0020764010382369] McLean C. Campbell C. Cornish F. (2003) African-Caribbean Interactions with Mental Health Services: Experiences and Expectations of Exclusion as (Re)productive of Health Inequalities. Social Science and Medicine 56(3), 657–669 12570981 10.1016/s0277-9536(02)00063-1

[bibr22-0020764010382369] Moffat J. Sass B. McKenzie K. Bhui K. (2009) Improving Pathways into Mental Health Care for Black and Ethnic Minority Groups: A Systematic Review of the Grey Literature. International Review of Psychiatry 21(5), 439–449 20374159 10.1080/09540260802204105

[bibr23-0020764010382369] Morgan C. Mallett R. Hutchinson G. Bagalkote H. Morgan K. Fearon P. Dazzan P. Boydell J. McKenzie K. Harrison G. Murray R. Jones P. Craig T. Leff J.; AESOP Study Group (2005a) Pathways to Care and Ethnicity. 1: Sample Characteristics and Compulsory Admission: Report from the AESOP Study. British Journal of Psychiatry 186, 281–289 15802683 10.1192/bjp.186.4.281

[bibr24-0020764010382369] Morgan C. Mallett R. Hutchinson G. Bagalkote H. Morgan K. Fearon P. Dazzan P. Boydell J. McKenzie K. Harrison G. Murray R. Jones P. Craig T. Leff J.; AESOP Study Group (2005b) Pathways to Care and Ethnicity. 2: Sources of Referral and Help Seeking. Report from the Aesop Study. British Journal of Psychiatry 186, 290–296 15802684 10.1192/bjp.186.4.290

[bibr25-0020764010382369] Norredam M. Garcia-Lopez A. Keiding N. Krasnik A. (2010) Excess Use of Coercive Measures in Psychiatry among Migrants Compared with Native Danes. Acta Psychiatrica Scandinavica 121(2), 143–151 19594483 10.1111/j.1600-0447.2009.01418.x

[bibr26-0020764010382369] Parkman S. Davies S. Leese M. Phelan M. Thornicroft G. (1997) Ethnic Differences in Satisfaction with Mental Health Services among Representative People with Psychosis in South London: PRiSM Study 4. British Journal of Psychiatry 171, 260–264 9337981 10.1192/bjp.171.3.260

[bibr27-0020764010382369] Pinto R. Ashworth M. Jones R. (2008) Schizophrenia in Black Caribbeans Living in the UK: An Exploration of Underlying Causes of the High Incidence Rate. British Journal of General Practice 58(551), 429–434 18505621 10.3399/bjgp08X299254 PMC2418996

[bibr28-0020764010382369] Raleigh V.S. Irons R.I. Hawe E. Scobie S. Cook A. Reeves R. Petruckevitch A. Harrison J. (2007) Ethnic Variations in the Experiences of Mental Health Service Users in England: Results of a National Patient Survey Programme. British Journal of Psychiatry 191, 304–312 17906240 10.1192/bjp.bp.106.032417

[bibr29-0020764010382369] Runyan C.W. Moracco K.E. Dulli L. Butts J. (2003) Suicide among North Carolina Women, 1989–93: Information from Two Data Sources. Injury Prevention 9(1), 67–72 12642563 10.1136/ip.9.1.67 PMC1730920

[bibr30-0020764010382369] Sass B. Moffat J. Bhui K. McKenzie K. (2009) Enhancing Pathways to Care for Black and Minority Ethnic Populations: A Systematic Review. International Review of Psychiatry 21(5), 430–438 20374158 10.1080/09540260802204121

[bibr31-0020764010382369] Singh S.P. (2007) Institutional Racism in Psychiatry: Lessons from Inquiries. Psychiatric Bulletin 31(10), 363–365

[bibr32-0020764010382369] Singh S.P. Greenwood N. White S. Churchill R. (2007) Ethnicity and the 1983 Mental Health Act. British Journal of Psychiatry 191, 99–105 17666492 10.1192/bjp.bp.106.030346

[bibr33-0020764010382369] Slade M. Powell R. Rosen A. Strathdee G. (2000) Threshold Assessment Grid (TAG): The Development of a Valid and Brief Scale to Assess the Severity of Mental Illness. Social Psychiatry and Psychiatric Epidemiology 35(2), 78–85 10784370 10.1007/s001270050011

[bibr34-0020764010382369] Vinkers D.J. de Vries S.C. van Baars A.W. Mulder C.L. (2010) Ethnicity and Dangerousness Criteria for Court Ordered Admission to a Psychiatric Hospital. Social Psychiatry and Psychiatric Epidemiology 45(2), 221–224 19396576 10.1007/s00127-009-0058-9

[bibr35-0020764010382369] Webber M. Huxley P. (2004) Social Exclusion and Risk of Emergency Compulsory Admission: A Case Control Study. Social Psychiatry and Psychiatric Epidemiology 39(12), 1000–1009 15583909 10.1007/s00127-004-0836-3

